# Inverted circadian variation of arterial pressure in a geriatric patient: an indicator of autonomic dysfunction

**DOI:** 10.1186/s12877-021-02059-3

**Published:** 2021-03-01

**Authors:** Siddhartha Lieten, Aziz Debain, Bert Bravenboer, Tony Mets

**Affiliations:** 1 Department of Geriatrics, Universitair Ziekenhuis Brussel, Laarbeeklaan 101, B-1090 Brussels, Belgium; 2 Frailty in Aging (FRIA) investigation group, Faculty of Medicine & Pharmacy, Vrije Universiteit Brussel (VUB), Laarbeeklaan 103, B-1090 Brussels, Belgium; 3 Departments of Endocrinology & Clinical Pharmacology, Universitair Ziekenhuis Brussel, Laarbeeklaan 101, B-1090 Brussels, Belgium

**Keywords:** Orthostatic hypotension, Nocturnal hypertension, Autonomic failure, 18-FDG PET brain CT scan

## Abstract

**Background:**

Orthostatic hypotension (OH) in geriatric patients frequently involves a component of autonomic failure (AF). The combination of OH with nocturnal hypertension (NHT) is indicative of AF, which is described as pure (PAF), when neurologic symptoms are absent, or as multisystem atrophy (MSA), when combined with motor disturbance (Parkinsonism or Parkinson disease).

**Case presentation:**

An 87-year-old man presented with long-lasting OH. He frequently fell, causing several fractures, and he developed heart failure. Blood pressure (BP) registration revealed a reversal of the day-night rhythm with NHT. An 18-FDG PET brain CT scan showed cerebellar hypometabolism, indicating MSA.

**Conclusions:**

This case demonstrates the use of continuous BP registration in geriatric patients with OH for diagnosing NHT. It illustrates the usefulness of 18-FDG PET brain CT scan to specify the nature of the AF. The case also illustrates the difficulty of managing the combination of OH and NHT.

## Background

Syncopes and falls are major geriatric issues, often related to blood pressure (BP) abnormalities. Orthostatic hypotension (OH) is a frequent finding in older people with a prevalence of more than 20 % above the age of 75 years [1, 2]. The origin of OH is complex, involving normal aging-related physiological changes, diseases, and drug induced etiologies.

Aging in itself results in a tendency towards OH by decreased cardiac output, bradycardia, and decreased baroreceptor sensitivity [3]. Postprandial intestinal vascular dilatation will induce peripheral blood pooling, as will the tendency for decreased muscular activity in old age. Subsequently, both will contribute to OH [3, 4].

The tendency for OH is often aggravated by pathological changes, such as anemia, common cardiac abnormalities (aortic valve stenosis, arrhythmias), diabetes mellitus (particularly in the postprandial phase after a carbohydrate rich meal), or changes resulting in a reduced circulating volume (e.g. adrenal failure). In addition, medication that can either produce hypovolemia (diuretics) or vasodilatation (anti-hypertensives, psychotropics, alpha-lytic drugs for prostatic disease) [5–7] frequently can provoke OH.

A component of autonomic failure (AF) is involved in many older patients with OH. It was observed in 27 % of a group of 100 patients referred for OH [5]. If no other neurologic symptoms are present, the condition is designated as Pure Autonomic Failure (PAF); if accompanied by motor disturbances, it is referred to as Multiple-System Atrophy (MSA). The motor component in MSA, described as “*Parkinsonism (bradykinesia with rigidity or tremor or both), usually with a poor or unsustained motor response to chronic levodopa therapy*” can be present in a various degrees [8]. Brain autonomic function is related to the activity of the dopaminergic system, which is known to decline with aging [9]. In recent animal experiments, epigenetic factors, which increase with aging, appear to be responsible for this decline [10]. In this context, it is not surprising that AF occurs more frequently in synucleopathies, the group of neurodegenerative diseases, consisting of Parkinson’s disease (PD) and Lewy body dementia [11].

AF is also observed in peripheral neuropathies. These become more frequent with aging and are mainly seen in the context of diabetes mellitus, chronic kidney disease, and vitamin B12 deficiency [12–14].

The concomitant presence of nocturnal hypertension (NHT) can complicate the management of OH. It is well known that BP has a circadian variation, with higher daytime and lower nighttime values, which drop on average by 10–20 % [15]. Some patients with OH can present NHT, often described as “non-dipping” or “reverse dipping” [16, 17]. At the origin of this NHT is the AF, resulting in insufficient sympathetic output while standing, but exerting residual activity in supine position [18, 19]. In a group of 52 OH patients (average age 77 years), NHT was present in 71 % of them [20]. The association between OH and NHT can be found in PD patients. In PD patients with OH, non-dipping was observed more frequently (78 % vs. 67 % in de non-OH patients) [21]. In another group of 188 PD patients, OH was observed frequently (30 %), while NHT was present in 25 % of them and both were associated [22].

Loss of a normal BP pattern with NHT is thought to contribute to the development of cardiovascular disease (CVD), chronic kidney disease, cognitive decline, and dementia [23–28]. Therefore, improved awareness of a non-dipping pattern of BP is important.

Here we describe a patient with multiple disorders, who illustrates the complex situation of OH combined with NHT.

## Case presentation

An 87-year-old man (weight 80.9 kg, height 1.79 m and BMI 25.3 kg/m^2^) presented with a syncope and fall at the emergency department. Upon arrival (at 4 p.m.) he was well oriented; BP (seated) was 168/59 mmHg with a regular pulse at 65/min (auscultatory). He had dyspnea (respiratory rate 15/min) with an arterial O_2_ saturation of 89 % (98 % after oxygen administration). His mouth mucosa was dry and he had bilateral pulmonary stasis, but no jugular tumescence. There was a slight degree of peripheral pitting edema and he had a urethral catheter. The neurologic examination was repeatedly normal; his MMSE score was 25/30. His basic ADL (Katz-scale) was 8/24; the instrumental ADL score (Lawton) was 15/27. He was mobile with a four-wheel walker under supervision from his wife. He was a previous smoker and admitted drinking three alcohol consumptions per day on average. His non-fasting glycemia was 107 mg/dL (fasting reference values 70–100 mg/dL) and follow-up revealed neither hyper- nor hypoglycemia; his hemoglobinemia was 12.6 g/dL (reference values: 13.0- 16.5 g/dL).

OH had been documented since approximately three years, with a systolic BP varying between 73 and 93 mmHg. An orthostatic test, two years before the present admission, had shown a BP of 184/111 mmHg and a heart rate (HR) of 73/min. in supine position. After standing one minute, BP was 98/70 mmHg and pulse rate 85/min; after three minutes 99/71 mmHg and 82/min.; after five minutes 98/63 mmHg and 78/min. He presented almost weekly syncopes and falls, mainly occurring after meals or physical effort. Convulsions, lasting ± 30 seconds, had been observed once. His medical history mentioned atrial fibrillation (with periods of slow ventricular response), bronchiectasis, colon diverticulosis, urge incontinence and urine retention, treated by trans-urethral catheter, and bilateral hip prosthesis for osteoporotic fractures after falls. Three months earlier, during a hospitalization for OH (86/53 mmHg), a discrete rigidity of the right arm and a slight bilateral tremor of the hands had been noted, suggesting an essential tremor or a beginning PD. It had been concluded that he suffered from severe OH, mainly due to sodium and fluid deficit, resulting in cerebral hypoperfusion. Several investigations had already been completed recently. A transthoracic cardiac ultrasound examination had shown no signs of cardiac amyloidosis, and a primary adrenal cortical failure was excluded.

His medication upon arrival consisted of rivaroxaban 15 mg q.d., amiodarone 200 mg q.d., bumetanide 1 mg q.d., spironolactone 25 mg q.d., fludrocortisone 0.1 mg b.i.d., finasteride 5 mg q.d., folic acid 4 mg q.d., calcium carbonate 1000 mg q.d., cholecalciferol 800 IE q.d., zolendronate 5 mg i.v. once a year, and paracetamol 1 g if necessary. He had been advised to wear elastic stockings, rise slowly, apply a mild anti-Trendelenburg position during the night, avoid large meals, and drink sufficiently.

Upon admission to the geriatric ward amiodarone and finasteride were stopped. He developed a pneumonia, empirically treated with piperacilline and tazobactam. This treatment was successful, but he developed heart failure, necessitating fludrocortisone to be stopped.

A CT scan of the brain showed slight cortico-subcortical and cerebellar atrophy, and some small lacunar infarctions in the basal ganglia. An EEG was normal. Given the history of syncopes, hypotension, and bradycardia, the cardiologist advised a coronaroangiography that showed only a moderate stenosis at two sites. An ultrasound examination of the neck showed no arterial stenosis, but revealed a multinodular thyroid goiter (thyroid hormone levels were found to be normal). An electromyography showed signs of length-dependent chronic axonal sensory-motoric polyneuropathy.

He had a calculated MDRD of 40ml/min, with normal renal ultrasound examination and no signs of renal artery stenosis. Continuous ECG registration confirmed a regular sinus rhythm with an average frequency of 52/min, (33–61/min) and a second degree A-V block Mobitz type II. Placing of a pacemaker normalized the heart rhythm, but syncopes and daytime OH persisted.

**Fig. 1:**
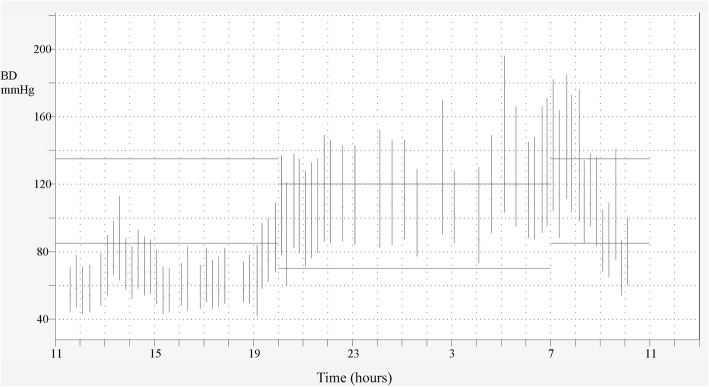
Circadian measurements of arterial blood pressure at the level of the upper arm (90217A Spacelabs Healthcare). Measurements every 15 minutes from 06 h - 22 h; every 30 minutes from 22 h - 06 h. Number of measurements performed: 91; successful measurements: 67 (86%); number of day-time (07-20 h) measurements: 42; number of night-time (patient supine, 20-07 h) measurements: 25. Horizontal bars indicate upper and lower limits of normal values: 135 and 85 mm Hg (day-time); 120 and 70 mm Hg (night-time), respectively systolic and diastolic

24-hours BP registration revealed a reversal of the day-night rhythm with hypotension during the day and hypertension during the night (see Fig. 1). The daytime average systolic, diastolic, and mean arterial pressures (StDev) were respectively 98 (33.7), 59 (19.2), and 72 (24.0) mm Hg; during the night these values were respectively 147 (17.0), 85 (8.8), and 106 (11.4) mm Hg. Trying to control the BP, bumetanide and spironolactone were stopped, and fludrocortisone (0.5 mg) was given in the morning and captopril (12.5 mg) in the evening. With this treatment, BP values varied between 80 and 180 mmHg with a tendency for improvement towards the end of his hospitalization that continued afterwards.

A PET brain scan showed no arguments for Lewy body disease. An 18 FDG PET CT brain scan demonstrated cerebellar hypometabolism, arguing in favour of MSA.

Due to the 2020 Covid 19 pandemic, we could not realize a follow-up 24 h BP registration. However, the patient reported a clear improvement with a significant reduction of symptomatic events.

## Discussion and conclusions

This geriatric patient with multiple morbidities, presented with heart failure, persistent OH, and frequent falls. He had no anemia, nor diabetes. Clearly, there was a therapeutic dilemma: the need to decrease the cardiac load, resulting in Na loss and insufficient circulating volume on the one hand, but on the other hand the need to raise his BP.

A remarkable feature in our patient was the association of daytime OH with NHT. Persistent NHT has been recognized in hypertensive older patients [29]. As detailed in the background section above, the combination of OH and NHT is suggestive of AF, in which sympathetic activity is insufficient and provokes OH, but where a residue of sympathetic activity often leads to hypertension in supine position and to NHT.

OH and NHT are more common in synucleotide diseases. Repeated neurological evaluation had not revealed PD in our patient and there were no arguments for Lewy body disease. Therefore, from a clinical point of view, he could be placed in the category of PAF. His history mentioned once the possibility of an essential tremor. Older patients presenting essential tremor do not show more AF [30], while in patients with restless legs syndrome, occurring in the context of PD, supine hypertension and NHT are more frequent [31]. Disturbed circadian BP rhythm with non-dipping has also been described in obstructive sleep apnea [32]; in our patient, a more detailed sleep observation has not been performed.

Besides OH, AF often implicates other aspects, including bladder dysfunction (both incontinence and retention, as in our patient), erectile dysfunction, xerostomia, pupillary abnormalities and dry eyes, transpiration, and gastro-intestinal complaints. Other aspects may be involved, as recent basic research in mice has shown that orthosympathetic denervation of the spleen leads to disturbed immunity, and that this pathway is under control of the amygdala and the paraventricular nucleus, and is linked to behavior [33]. Hence, the complete spectrum of consequences of AF remains to be further investigated.

The orthostatic test criteria to identify OH remain valid for geriatric patients: ≥20 mmHg systolic, or ≥ 10 mmHg diastolic BP decrease within five minutes of standing, after a supine rest of five minutes [34]. Often, the expected rise in heart rate will be blunted. In OH patients, NHT was found to be associated (in 65 %) with an insufficient heart rhythm increase upon standing [20]. Recently, the Autonomic Disorders Consortium reported a value lower than 0.492 bpm/mmHg in the ratio Δ HeartR/Δ Systolic BP as diagnostic to identify neurogenic OH, with a sensitivity 91.3 % and a specificity 88.4 %) [12]. In our patient, the ratio Δ HeartR/Δ Systolic BP was 0.140 after 1 minute, 0.106 after 3 minutes, and 0.058 after 5 minutes of standing, confirming the usefulness of this ratio.

Valsalva manoeuver or the head-up tilt test are not common practice for ambulatory geriatric patients [35]. NHT is more difficult to observe and requires ambulatory BP monitoring with nocturnal registration.

Additional testing for AF includes 18-FDG PET CT, which in case of MSA will show cerebellar hypometabolism, as in our patient [36]. Therefore, rather than the first hypothesis of PAF, we can conclude that our patient presents MSA, despite the absence of obvious clinical neurological symptoms. In the differential diagnosis procedure, electromyography can document peripheral neuropathy, which was also present in our patient, and may have contributed to the severity of his problem. Evaluation of the cardiac and cerebral circulation is useful, as OH can result in impaired organ perfusion.

Treatment for OH will mainly consist of measures intended to alleviate BP falls, similar to those followed by our patient: wearing elastic stockings, rising slowly, avoid large meals, and drink sufficiently. Applying mild anti-Trendelenburg position will not be advisable in case of NHT. Pharmaceutical treatment will mainly consist of fludrocortisone, but NHT might be worsened. NHT is reported to react to transdermal nitroglycerin (0.025 to 0.1 mg/h), applied in the evening [37]. In our patient nighttime antihypertensive treatment was preferred. A weakness in our case report is that no follow-up 24 h BP record is available to better document the treatment effect. In mice, and still at the experimental level, treatment with a sodium-glucose cotransporter 2 (SGLT2) inhibitor has shown to normalize circadian BP rhythm [38, 39]. To our knowledge no human studies have been performed.

In the prevention and treatment of OH for geriatrics patients there has been insufficient focus on intensifying muscle activity. Contracting leg muscles exert a pump function and strengthen the venous return. Intensive activity also leads to physiologic changes that upgrade homeostasis [40].

With this case report, we draw the attention to the complex situation of AF that results in simultaneous OH and NHT. The diagnostic approach is rather complicated for geriatric patients, as it needs continuous BP registration and cerebral imaging. Presently, preventive and therapeutic options have not been well investigated. Therefore, further studies are needed.

## Data Availability

All data generated or analysed during this study are included in this published article [and its supplementary information files].

## Abbreviations

AF: Autonomic failure
BP: Blood pressure
CVD: Cardiovascular disease
HRV: Heart rate variability
NHT: Nocturnal hypertension
OH: Orthostatic hypotension
PAF: Pure autonomic failure
MSA: Multisystem atrophy
